# Supplementary figures and images for: A dual model node based optimization algorithm for simultaneous escape routing in PCBs
Source: PeerJ Comput Sci. 2021 Apr 16;7:e499. doi: 10.7717/peerj-cs.499 (PMC8056246; doi:10.7717/peerj-cs.499)

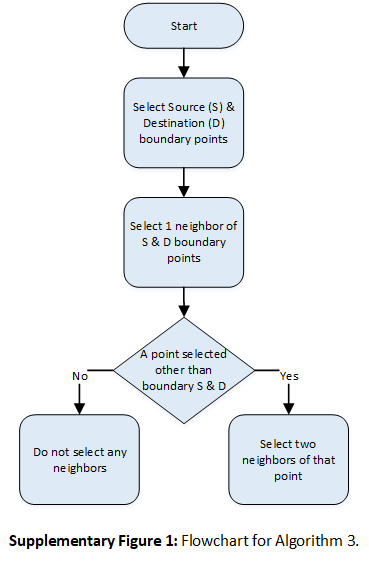

Supplement: Supplemental Information 1 [file peerj-cs-07-499-s001.png]

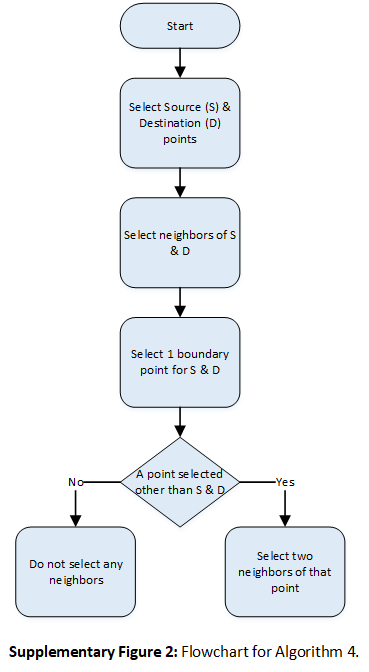

Supplement: Supplemental Information 2 [file peerj-cs-07-499-s002.png]
